# Political Ideology and HPV Vaccine Awareness: Sex Differences and Nursing Implications Using the Health Information National Trends Survey (HINTS)

**DOI:** 10.1155/nrp/4233079

**Published:** 2026-03-28

**Authors:** Soojung Jo, Dongjuan Xu, Monica L. Kasting, Nicole Adams

**Affiliations:** ^1^ School of Nursing, Purdue University, West Lafayette, Indiana, USA, purdue.edu; ^2^ Department of Public Health, Purdue University, West Lafayette, Indiana, USA, purdue.edu; ^3^ Regenstrief Center for Healthcare Engineering, Purdue University, West Lafayette, Indiana, USA, purdue.edu

**Keywords:** cancer vaccines, human papillomavirus viruses, papillomavirus vaccines, politics, vaccines

## Abstract

**Background:**

This study aims to investigate the relationship between political ideology and the awareness of human papillomavirus (HPV) and the HPV vaccine and the interaction with sex and race/ethnicity.

**Methods:**

This cross‐sectional study is a secondary analysis of Health Information National Trends Survey data, based on responses from 3113 American households. The analysis used listwise deletion. Data included awareness of HPV and the HPV vaccine, political ideology, and demographic characteristics. Multivariable logistic regression models with interaction terms were used to examine the associations between political ideology, sex, race/ethnicity, and HPV awareness outcomes.

**Results:**

Moderate and conservative populations were less likely to be aware of HPV and the HPV vaccine compared to liberal population. However, these differences were only observed among males. Black, Hispanic, Asian, and other populations were less aware of HPV and the HPV vaccine than the White populations. Younger age, higher educational level, and higher income were associated with awareness.

**Conclusions:**

The results of this study highlight the need for tailored outreach strategies for conservative males and racial/ethnic minority populations by tailoring approaches to increase awareness of the dangers of HPV and the benefits of vaccination. Nurses should play a critical role in delivering culturally appropriate education and strong vaccine recommendations to improve awareness and uptake by targeting conservative males and underserved racial and ethnic minority groups who consistently demonstrate lower levels of HPV‐related knowledge.

## 1. Introduction

In the United States (U.S.), human papillomavirus (HPV) is the most common sexually transmitted infection and is associated with several health risks. These include the development of various cancers such as anal, cervical, vaginal, vulvar, penile, and oropharyngeal cancers in both males and females (US Centers for Disease Control and Prevention [1]; US National Cancer Institute [2, 3]). HPV‐associated cancer accounts for nearly 50,000 individual cancer cases in the US [1]. Out of these cases, approximately 98% of cervical cancers, 90% of anal cancers, and almost 70% of oropharyngeal cancers are attributable to HPV [1, 4, 5].

The HPV vaccine has demonstrated efficacy in preventing cancers, showing potential to eliminate nearly 100% of infections caused by seven HPV types causing cancers [2]. However, HPV vaccine rates remain low. In 2022, only 62.6% of adolescents aged 13 to 17 were up‐to‐date with their HPV vaccinations [6]. This falls below the Healthy People 2030 goal of 80% coverage [7] and is lower than vaccine rates for measles, mumps, and rubella (91.2%) and tetanus, diphtheria, and pertussis (89.9%) [6]. Such insufficient coverage threatens to perpetuate the burden of HPV‐related diseases in the US.

## 2. Background

Several studies have reported the associated factors of the vaccine uptake in general including knowledge, provider recommendations, and some belief factors such as positive attitudes towards vaccination [8]. Studies highlight the influential role of political ideology, especially after the COVID‐19 pandemic. The contentious debate surrounding the COVID‐19 vaccine provides a clear illustration. In a study of the European population, people on the extreme ends of the ideological spectrum both demonstrated a higher level of skepticism toward the COVID‐19 vaccine [9]. One study in the U.S. found that the more politically conservative people are more likely to refuse COVID‐19 vaccination [10]. This is consistent with findings of a prepandemic study which evaluated intent to receive vaccines such as pertussis, measles, and influenza for children. They found that conservatives had a lower intent to vaccinate, although this was mediated by a distrust of the government [11]. These findings highlight the principle that political ideology acts as a powerful determinant in shaping health attitudes and behaviors. To explain the mechanisms behind these polarized health responses, the Ideological Health Spirals Model posits that political orientation and psychological traits are the drivers for social interactions and media selection and eventually impact health behavior [12]. Supporting this framework, a recent study found that online health information seeking is associated with greater HPV awareness [13], suggesting that media selection shaped by political orientation may indirectly influence HPV vaccine awareness. This process reinforces communication discrepancies, resulting in distinct gaps in attitudinal, normative, and efficacy‐related health beliefs across ideological groups, ultimately driving different health behaviors, such as HPV vaccination [12].

While existing literature has examined the association between political ideology and vaccine attitudes, there is a need for further investigation specific to HPV awareness. HPV vaccination is heavily influenced by awareness of HPV and the HPV vaccine [14]. HPV vaccination behavior presents unique challenges due to its perceived ties with sexual activity, adding layers of complexity to how political ideology might shape HPV awareness and acceptance. Recognizing the importance of HPV awareness is vital, as it is identified as a key factor in increasing HPV vaccine uptake, thus optimizing the vaccine’s potential benefits [14].

One emerging area of interest in recent years is the influence of political ideology on vaccine attitudes, particularly regarding the HPV vaccine. Some studies indicate that individuals often erroneously believe that receiving the HPV vaccination will result in an increase in sexual promiscuity, leading to vaccine refusal [15]. However, research has shown that HPV vaccination does not increase sexual activity [16]. This vaccine refusal is further compounded by some parents’ reluctance to engage in discussions with their children about sex, making the topic of HPV vaccination even more challenging to broach [17, 18]. Conservatives tend to perceive less need for sex education and generally avoid such discussions [19], which could further impact their HPV vaccine uptake. Given the complexities identified, the findings of this study hold significant relevance for nursing practice. Nurses, particularly in primary care and school health settings, are often the frontline educators and trusted conduits of health information for patients and parents. Understanding how political ideology shapes HPV awareness is crucial for nurses to tailor their communication strategies, address ideological barriers, and effectively counsel parents on the vaccine’s benefits, ultimately improving uptake rates. Therefore, this study aims to investigate (1) the relationship between political ideology and the awareness of HPV and the HPV vaccine and (2) whether the relationship varies by sex and by race/ethnicity.

## 3. Methods

### 3.1. Design

This study utilized a cross‐sectional design using secondary data. This secondary data analysis utilized the public data set from the Health Information National Trends Survey (HINTS) which was conducted by the US National Cancer Institute, a national initiative focused on gathering insights related to cancer knowledge, perceptions, and preventive behaviors [20]. HINTS 5 received an Exempt determination from the National Institutes of Health Office of Human Subjects Research (Exempt #13204).

### 3.2. Instrument

Outcome variables. In our study, we analyzed the primary outcome from the following: “Are you familiar with HPV?” HPV refers to human papillomavirus. It is distinct from HCV, HIV, HSV, or herpes” for assessing HPV awareness, and “There’s a vaccine against HPV infection known as the HPV shot or cervical cancer vaccine, GARDASIL. Prior to today, were you aware of the cervical cancer vaccine or HPV shot?” for assessing HPV vaccine awareness. Answers to both questions were either “yes” or “no.”

Independent variables. We determined political ideology using the following question: “Thinking about politics these days, how would you describe your own political viewpoint?” Respondents selected from seven options, which were from ‘Very Liberal,’ ‘Liberal,’ ‘Somewhat liberal,’ ‘Moderate,’ ‘Somewhat conservative,’ ‘Conservative,’ and ‘Very Conservative.’ For the purpose of this analysis, responses were consolidated into three categories: ‘Very Liberal,’ ‘Liberal,’ and ‘Somewhat Liberal’ were grouped as ‘Liberal;’ ‘Moderate’ remained as its own category; and ‘Somewhat Conservative,’ ‘Conservative,’ and ‘Very Conservative’ were categorized as ‘Conservative.’

Sex at birth was categorized into two groups: females and males. In terms of race/ethnicity, respondents were categorized as White (non‐Hispanic White), Black (non‐Hispanic Black), Asian (non‐Hispanic Asian), other (non‐Hispanic other, including American Indian/Alaska Native, Native Hawaiian/Pacific Islander, and multiracial individuals), and Hispanic (of any race).

Control variables. Besides sex at birth and race/ethnicity, we included demographic variables such as age, marital status, educational level, income, employment status, geographical location, and insurance status. We categorized age into groups: 18–26, 27–35, 36–45, 46–64, and above 65. We chose these age categories to be congruent with Food and Drug Administration licensure for the HPV vaccine. The vaccine is licensed for those up to age 45. However, people over the age of 45 may have age‐eligible children for whom they are the medical decision‐makers. Therefore, we included those over age 45 in the study sample and kept them in a separate category. Marital statuses were grouped into three categories: married/partnered, divorced/widowed/separated, and single (never married). Educational levels were divided into four categories: less than high school, high school graduate, some college, and college graduate or above. Income classifications were based on the 2020 US median income of $67,521, leading to the following brackets: < $20,000, $20,000–$49,999, $50,000–$74,999, $75,000–$99,999, and ≥ $100,000. Employment was binary: either employed (yes) or not employed (no). Geographical location was defined as either metropolitan or nonmetropolitan, using the U.S. Department of Agriculture’s 2013 Rural‐Urban Continuum Codes used for recruitment stratification. Insurance was coded as either “yes”, indicating possession of any form of insurance or “no”.

### 3.3. Sample

The data utilized in this research is derived from the HINTS 5 Cycle 4 conducted in 2020 (data collection spanned from February 24 to June 15, 2020). HINTS released the newer cycle in the 2022, but the main variable, political ideology, was only included in 2020 survey. HINTS employed stratified random sampling based on addresses, categorizing them into two segments: areas with a high density of minority populations and those with a lower density. The strata were based on the census tract‐level demographic data extracted from the 2014–2018 American Community Survey. The sampling methodology involved a two‐stage process: the initial stage selected the housing address, followed by a second stage in which one adult was selected from the sampled household. The survey was mailed to American households, and responses were sent back in the same manner. The survey was sent to 15,347 households, and 3865 were returned, representing a 37% response rate. The dataset is publicly available through the HINTS portal.

Out of the 3865 surveys returned, there were 3418 in which respondents reported their political ideology. We used only the records that answered all variables for the analysis, resulting in 3113 records in our final sample (91.08%). The primary reason for record exclusion was missing data, most commonly for race/ethnicity items (185 cases, 4.8%). Since the missing rate was below 10% [21], listwise deletion was applied in the analysis.

### 3.4. Analytic Strategy

We described the sample using descriptive statistics. We had two outcome variables: HPV awareness and the HPV vaccine awareness. We performed the same data analyses for each outcome separately. We used chi‐square tests to compare the group differences on HPV awareness and the HPV vaccine awareness in terms of political ideology, sex, and race/ethnicity. We performed multivariable logistic regression models to investigate the relationship between political ideology and outcome variables. We used interaction terms (i.e., political ideology ∗ sex and political ideology ∗ race/ethnicity) to determine if the relationship between political ideology and outcome variables varied by sex and race/ethnicity. The interaction term political ideology ∗ race/ethnicity was not statistically significant in any of the models. To be parsimonious, we only reported estimates of the political ideology ∗ sex interaction term. We adjusted all logistic regression models by controlling for variables: age, marital status, education level, income, employment, location, and insurance status. For some variables of interest (e.g., political ideology), if there was a statistically significant difference, we examined different reference categories to explore the differences between all of the groups. All analyses utilized jackknife replicate weights and calculation of standard error estimates to account for complex survey design as per the guidance from HINTS. We used Stata Version 18.0 (StataCorp, College Station, Texas) and a *p* value < 0.05 (two‐sided) for establishing statistical significance.

## 4. Results

### 4.1. Demographic Characteristics

Table 1 and Supporting Table 1 display the characteristics of the sample, both in unweighted and weighted forms. The average age, when adjusted for survey weights, was 47.4 years (S.E. = 0.32). The largest percentage was 46–64 years old (36.1%). The majority were White (63.8%), and the sample was fairly evenly distributed by sex and political ideology. About two‐thirds of the respondents were aware of HPV (67.4%) and the HPV vaccine (63.6%). Over half (57.0%) were aware of both HPV and the HPV vaccine, with 10.4% of the respondents being aware of HPV only (and not the HPV vaccine), 6.6% being aware of the HPV vaccine (and not HPV itself), and 26.0% being not aware of either.

**TABLE 1:** Characteristics of study participants (weighted population *n* = 213, 549, 306).

	Percent or mean	95% CI	
		Lower	Upper
Age (years)	47.36	46.71	48.01
18–26	14.89%	12.73%	17.35%
27–35	14.05%	12.01%	16.36%
36–45	16.61%	14.67%	18.75%
46–64	36.09%	33.87%	38.37%
≥ 65	18.36%	17.59%	19.16%
Sex			
Male	49.70%	48.58%	50.81%
Female	50.30%	49.19%	51.42%
Race/ethnicity			
White	63.84%	62.81%	64.85%
Black	10.71%	10.06%	11.40%
Hispanic	17.03%	16.35%	17.74%
Asian	5.46%	4.84%	6.15%
Other	2.96%	2.36%	3.69%
Marital Status			
Married/partnered	55.13%	53.80%	56.45%
Divorced/widowed/separated	13.34%	12.33%	14.41%
Single, never been married	31.53%	30.33%	32.76%
Education level			
Less than high school	7.07%	5.55%	8.95%
High school graduate	21.02%	19.02%	23.17%
Some college	39.71%	37.78%	41.68%
College graduate or more	32.20%	31.46%	32.95%
Income			
< $20,000	13.29%	11.59%	15.19%
$20,000–$49,999	23.47%	21.11%	26.00%
$50,000–74,999	18.86%	15.99%	22.10%
$75,000–$99,999	12.70%	10.83%	14.83%
≥ $100,000	31.69%	28.72%	34.81%
Employment status			
Yes	61.64%	58.86%	64.35%
No	38.36%	35.65%	41.14%
Location			
Metropolitan	88.54%	86.58%	90.25%
Nonmetropolitan	11.46%	9.75%	13.42%
Insurance			
Yes	91.01%	90.06%	91.87%
No	8.99%	8.13%	9.94%
Political ideology			
Liberal	29.97%	27.58%	32.48%
Moderate	36.76%	33.83%	39.79%
Conservative	33.27%	30.42%	36.24%
HPV awareness			
Yes	67.37%	64.10%	70.47%
No	32.63%	29.53%	35.90%
HPV vaccine awareness			
Yes	63.57%	60.67%	66.37%
No	36.43%	33.63%	39.33%

Abbreviation: CI, confidence interval.

### 4.2. Group Comparison (Weighted, Unadjusted Results)

The analysis of the group comparison shows that HPV and the HPV vaccine awareness varied by political ideology, sex, and race/ethnicity (Table 2). Regarding political ideology, a larger percentage of liberals were aware of HPV (liberals: 78.3%; moderates: 64.7%; and conservatives: 60.5%) and the HPV vaccine (liberals: 73.7%; moderates: 59.1%; and conservatives: 59.4%). A larger percentage of females were aware of HPV (females: 74.4%; males: 60.2%) and the HPV vaccine (females: 75.2%; males: 51.8%) than males. Whites had the highest percentage of being aware of HPV (72.4%) and the HPV vaccine (70.3%), and Asians had the lowest awareness of HPV (45.0%) and the HPV vaccine (47.5%).

**TABLE 2:** HPV awareness and HPV vaccine awareness by political ideology, sex, and race and ethnicity, respectively.

	HPV awareness		HPV vaccine awareness	
	Percent (95% CI)	*p* value	Percent (95% CI)	*p* value
Political ideology				
Liberal	**78.34%** (73.53%, 82.48%)	**< 0.001**	**73.70%** (67.98%, 78.72%)	**< 0.001**
Moderate	**64.66%** (58.86%, 70.05%)		**59.06%** (54.65%, 63.33%)	
Conservative	**60.47%** (55.94%, 64.84%)		**59.42%** (55.05%, 63.66%)	
Sex				
Female	**74.41%** (70.74%, 77.76%)	**< 0.001**	**75.20%** (72.23%, 77.94%)	**< 0.001**
Male	**60.24%** (55.20%, 65.07%)		**51.80%** (47.01%, 56.56%)	
Race/ethnicity				
White	**72.43%** (69.31%, 75.35%)	**< 0.001**	**70.31%** (67.18%, 73.25%)	**< 0.001**
Black	**62.33%** (52.65%, 71.11%)		**58.25%** (48.29%, 67.58%)	
Hispanic	**59.91%** (51.91%, 67.41%)		**49.15%** (41.82%, 56.51%)	
Asian	**44.96%** (32.10%, 58.53%)		**47.49%** (34.62%, 60.70%)	
Other	**60.58%** (44.88%, 74.36%)		**50.15%** (30.16%, 70.10%)	

*Note:* Bold values indicate statistically significant results.

### 4.3. HPV and the HPV Vaccine Awareness by Political Ideology, Sex, and Race/Ethnicity (Weighted, Adjusted Results)

**FIGURE 1:**
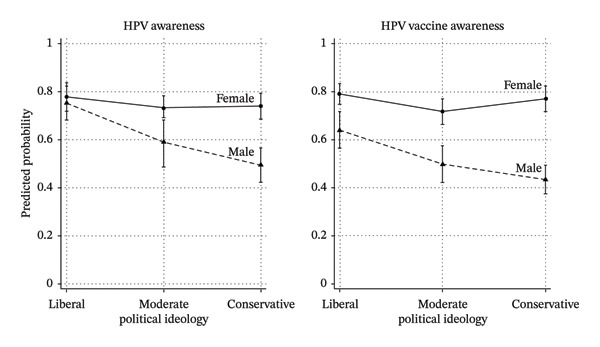
The predicted probability of HPV awareness and HPV vaccine awareness by political ideology and sex, respectively. Notes: covariates included in the model: race/ethnicity, age, marital status, education level, income, employment status, location, and insurance.

After controlling for sociodemographic variables, the logistic regression models confirm our earlier bivariate group comparisons, indicating significant differences in political ideology and sex concerning awareness of HPV and the HPV vaccine (Tables 3 and 4 and Figure 1). Compared with liberals, moderates and conservatives were less likely to be aware of HPV and the HPV vaccine. There were no significant differences between moderates and conservatives in terms of HPV awareness and the HPV vaccine awareness (Table 4). Females were more likely to be aware of HPV and the HPV vaccine than males. Moreover, we found significant interactions between sex and political ideology (Table 3).

**TABLE 3:** Factors associated with HPV awareness and HPV vaccine awareness, respectively.

	HPV awareness					HPV vaccine awareness				
	aOR	SE	*p* value	95% CI		aOR	SE	*p* value	95% CI	
				Lower	Upper				Lower	Upper
Political Ideology (ref. liberal)										
Moderate	**0.40**	0.15	**0.019**	0.19	0.85	**0.48**	0.14	**0.015**	0.27	0.86
Conservative	**0.25**	0.07	**< 0.001**	0.15	0.44	**0.35**	0.09	**< 0.001**	0.20	0.60
Sex (ref. male)										
Female	1.18	0.36	0.588	0.64	2.17	2.56	0.61	**< 0.001**	1.59	4.13
Political Ideology ∗ Sex (ref. liberal male)										
Moderate female	1.84	0.76	0.145	0.81	4.20	1.28	0.49	0.518	0.60	2.75
Conservative female	**3.07**	1.08	**0.003**	1.51	6.24	**2.51**	0.78	**0.004**	1.35	4.67
Race/ethnicity (ref. White)										
Black	**0.54**	0.13	**0.012**	0.33	0.87	**0.58**	0.15	**0.035**	0.35	0.96
Hispanic	**0.41**	0.10	**0.001**	0.24	0.68	**0.29**	0.06	**< 0.001**	0.19	0.45
Asian	**0.14**	0.04	**< 0.001**	0.08	0.27	**0.20**	0.08	**< 0.001**	0.09	0.44
Other	**0.39**	0.14	**0.011**	0.19	0.80	**0.28**	0.13	**0.011**	0.11	0.74
Age (ref. 18–26 years)										
27–35 years	1.01	0.36	0.987	0.49	2.07	0.55	0.20	0.108	0.26	1.15
36–45 years	1.24	0.44	0.553	0.61	2.52	0.56	0.23	0.157	0.25	1.26
46–64 years	**0.52**	0.16	**0.043**	0.27	0.98	**0.31**	0.11	**0.001**	0.15	0.61
≥ 65 years	**0.17**	0.05	**< 0.001**	0.09	0.31	**0.12**	0.05	**< 0.001**	0.06	0.27
Marital Status (ref. married/partnered)										
Divorced/widowed/separated	1.11	0.19	0.560	0.78	1.56	1.01	0.17	0.932	0.73	1.42
Single, never been married	1.05	0.26	0.835	0.64	1.72	0.75	0.19	0.259	0.46	1.24
Education level (ref. < high school)										
High school graduate	1.17	0.52	0.727	0.48	2.84	1.94	0.73	0.085	0.91	4.12
Some college	1.99	0.86	0.118	0.83	4.75	**4.34**	1.44	**< 0.001**	2.22	8.47
College graduate or more	2.66	1.30	0.051	1.00	7.11	**4.72**	1.81	**< 0.001**	2.18	10.19
Income (ref. < $20,000)										
$20,000–$49,999	1.41	0.29	0.107	0.93	2.13	0.96	0.18	0.817	0.66	1.39
$50,000–$74,999	1.28	0.44	0.484	0.64	2.56	0.74	0.14	0.122	0.50	1.09
$75,000–$99,999	1.98	0.74	0.075	0.93	4.21	1.41	0.42	0.258	0.77	2.55
≥ $100,000	**2.32**	0.87	**0.029**	1.09	4.91	**2.19**	0.55	**0.003**	1.32	3.64
Employment status (ref. no)										
Yes	0.90	0.17	0.566	0.61	1.31	0.98	0.18	0.897	0.68	1.41
Location (ref. metropolitan)										
Nonmetropolitan	0.92	0.29	0.801	0.50	1.72	1.21	0.46	0.616	0.57	2.58
Insurance (ref. no)										
Yes	1.39	0.56	0.418	0.62	3.15	1.03	0.33	0.933	0.54	1.97

*Note:* Bold values indicate statistically significant results.

Abbreviations: aOR, adjusted odds ratio; CI, confidence interval; SE, standard error.

**TABLE 4:** HPV awareness and HPV vaccine awareness by political ideology and sex, receptively.

	HPV awareness				HPV vaccine awareness			
	aOR	*p* value	95% CI		aOR	*p* value	95% CI	
			Lower	Upper			Lower	Upper
Political Ideology								
Moderate vs. Liberal	**0.90**	**0.009**	0.83	0.97	**0.90**	**0.001**	0.84	0.96
Conservative vs. Liberal	**0.86**	**< 0.001**	0.81	0.92	**0.89**	**0.001**	0.84	0.95
Conservative vs. Moderate	0.96	0.228	0.89	1.03	0.99	0.855	0.94	1.05
Sex								
Female vs. Male	**1.15**	**< 0.001**	1.09	1.22	**1.27**	**< 0.001**	1.22	1.32

*Note*: Covariates included in the model: race/ethnicity, age, marital status, education level, income, employment status, location, and insurance. Bold values indicate statistically significant results.

Abbreviations: aOR, adjusted odds ratio; CI, confidence interval; SE, standard error.

The differences in sex became more pronounced along the political continuum from liberal to conservative (Figure 1, and Table 5). In terms of HPV awareness, the difference between males and females was not statistically significant among liberals or among moderates, but it was statistically significant among conservatives (females 73.9%, males 49.6%). In terms of HPV vaccine awareness, the difference between males and females was 15% among liberals, 22% among moderates, and 34% among conservatives (females 77.0%, males 43.4%). Furthermore, the differences in HPV and the HPV vaccine awareness based on political ideology were observed only among males, with no significant differences found among females. Compared with conservative males, liberal males reported a higher likelihood of being aware of HPV (liberal males 75.3%, conservative males 49.6%) and the HPV vaccines (liberal males 64.0%, conservative males 43.4%).

**TABLE 5:** The predicted probability of HPV awareness and HPV vaccine awareness by political ideology and sex, respectively.

Political ideology and sex	HPV awareness			HPV vaccine awareness		
	Predicted probability (%)	95% CI		Predicted probability (%)	95% CI	
		Lower (%)	Upper (%)		Lower (%)	Upper (%)
Liberal Female	77.76	71.82	83.70	79.01	74.72	83.31
Liberal Male	75.26	68.25	82.27	63.96	56.33	71.60
Moderate Female	73.14	67.99	78.29	71.72	66.37	77.07
Moderate Male	58.97	48.69	69.25	49.83	42.39	57.28
Conservative Female	73.89	68.48	79.30	77.03	71.97	82.08
Conservative Male	49.56	42.33	56.78	43.43	37.58	49.27

*Note:* Covariates included in the model: race/ethnicity, age, marital status, education level, income, employment status, location, and insurance.

**FIGURE 2:**
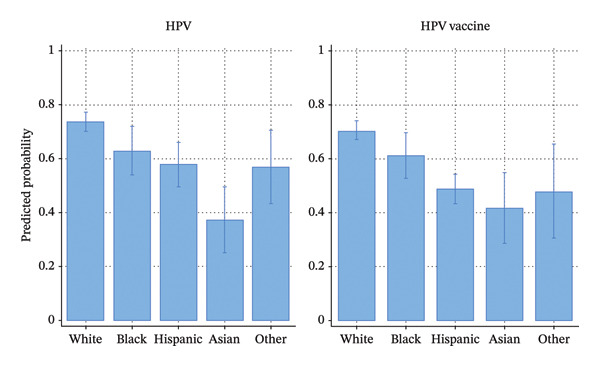
The predicted probability of HPV awareness and HPV vaccine awareness by race and ethnicity, respectively. Notes: covariates included in the model: political ideology, sex, age, marital status, education level, income, employment status, location, and insurance.

There were differences in HPV awareness and the HPV vaccine awareness by race/ethnicity (Tables 3 and 6, and Figure 2). Black, Hispanic, Asian, and other populations had lower odds of being aware of HPV compared to the White population. Compared to the White population, Asians had the lowest odds of HPV awareness (aOR = 0.14, *p* < 0.001). Furthermore, post hoc comparisons confirmed Asians had significantly lower odds than Blacks (aOR = 0.27, *p* = 0.001), Hispanics (aOR = 0.35, *p* = 0.005), and others (aOR = 0.37, *p* = 0.012). Regarding the HPV vaccine awareness, all non‐White populations (Black: aOR = 0.58, *p* = 0.035; Hispanic: aOR = 0.29, *p* < 0.001; Asian: aOR = 0.20, *p* < 0.001; and other: aOR = 0.28, *p* = 0.011) had lower odds of being aware of HPV vaccine compared to the White population. Additionally, the Black population had higher odds of being aware of the HPV vaccine than the Asian population (aOR = 2.87, *p* = 0.005) and Hispanics (aOR = 1.97, *p* = 0.027). Except for the above significant differences, there were no other significant differences in HPV and the HPV vaccine awareness among other racial/ethnic groups.

**TABLE 6:** The predicted probability of HPV awareness and HPV vaccine awareness by race/ethnicity, respectively.

Race and ethnicity	HPV awareness			HPV vaccine awareness		
	Predicted probability	95% CI		Predicted probability	95% CI	
		Lower	Upper		Lower	Upper
White	73.66	70.11	77.22	70.51	67.03	73.98
Black	62.95	53.92	71.98	61.29	52.84	69.74
Hispanic	57.73	49.50	65.96	48.70	43.22	54.18
Asian	37.18	25.14	49.22	41.65	28.58	54.72
Other	56.89	43.32	70.47	47.97	30.49	65.44

*Note:* Covariates included in the model: political ideology, sex, age, marital status, education level, income, employment status, location, and insurance.

### 4.4. Other Factors Associated With HPV and the HPV Vaccine Awareness (Weighted, Adjusted Results)

We found that individuals aged 46–64 years and 65 years and older had lower odds of being aware of HPV and the HPV vaccines compared to those aged 18–26 years. Individuals with some college education and above reported higher odds of the HPV vaccine awareness than those with less than a high school education. Similarly, individuals with some college education and above had higher odds of being aware of both HPV and the HPV vaccine than high school graduates. There were no significant differences between those with less than a high school education and high school graduates in terms of awareness of HPV or the HPV vaccine. Individuals with income equal to or greater than $100,000 had higher odds of being aware of HPV and the HPV vaccine than those with income less than $20,000. There were no statistically significant differences based on marital status, employment status, geographical location, or insurance.

## 5. Discussion

This study analyzed the relationship between political ideologies and awareness of HPV and the HPV vaccine and its relationship with sex and race/ethnicity and other demographic variables. Among the whole population, we see there are differences in HPV and the HPV vaccine awareness based on political ideology. However, on further inspection, we found these differences stemmed from the differences among males, as we did not find significant differences among females. In general, females were more likely to be aware of HPV and the HPV vaccine than males. In terms of race/ethnicity, Black, Hispanic, Asian, and other racial/ethnic populations are less likely to be aware of HPV and the HPV vaccine. Although there was no interaction between race/ethnicity and political ideology, race/ethnicity was still a significant variable while controlling for other variables.

From this study, we found generally low awareness of HPV at 67.37% and the HPV vaccine at 63.57% across the population. This trend aligns with global observations; for example, HPV vaccine awareness was reported at 69.9% in Italy [14] and 76.6% among European parents of adolescents. The findings underscore the disparity in vaccine awareness across different regions and populations. Given that only 62.6% of adolescents aged 13–17 were up‐to‐date with their HPV vaccinations [6], and considering that vaccine acceptance is closely related to awareness [22], this result reemphasizes the importance of increasing HPV awareness.

The significant result of the interaction with sex and political ideology was showing that HPV awareness had variation by political ideology only among males. Healthcare Providers (HCPs) play a crucial role in informing parents about HPV. Studies have shown that approximately half of the parents learned about HPV from providers [23, 24]. Additionally, HCP recommendations are a significant driver of vaccination [25, 26]. However, males tend to receive HPV vaccination recommendations from their HCPs less frequently compared to females [27]. Since the introduction of the HPV vaccine, its promotion has primarily focused on preventing cervical cancer [28]. This focus on cervical cancer has led to a higher level of awareness and knowledge about HPV among females compared to males [29, 30]. Although the HPV vaccine is recommended in primary health settings and pediatrics, the emphasis in obstetrics and gynecology practices may reduce the differences in HPV awareness among females, irrespective of their political ideologies, thereby enhancing overall awareness among females. Understanding these factors can help develop targeted strategies to increase HPV vaccine awareness and vaccination. Future qualitative research is needed to explore the mechanisms behind lower HPV awareness among conservative males and to better understand their information‐seeking behaviors and trusted sources.

Our study also examined the race/ethnicity disparities that persist in HPV awareness. A previous meta‐analysis has identified Black and Hispanic populations were less likely to complete the HPV vaccine series than Whites [31]. The results from our study reaffirm that this lower rate of vaccination might be deeply intertwined with a lack of awareness or access to information among race/ethnicity minority populations. Moreover, Asian Americans’ HPV awareness in our study was significantly lower than all other races/ethnicities. It is essential to interpret such findings within cultural and societal contexts. For example, prior research has emphasized a potential hesitancy among some Asian American parents and young adults to receive/recommend the HPV vaccine due to incorrect knowledge or false beliefs about the social stigma associated with the vaccination for HPV [32, 33] or low literacy and knowledge [34]. Additionally, emerging literature suggests that broader structural factors affecting Asian American communities, such as language barriers in health communication, underrepresentation in targeted public health outreach, and the tendency to treat diverse Asian subgroups as a single homogeneous population, may further limit access to accurate HPV‐related information and contribute to persistent disparities [35]. Our findings highlight the urgent need to enhance HPV awareness in race/ethnicity minority populations, specifically targeting Asian American communities, by targeting approaches to align with the diverse cultural and racial identities of each community.

Other factors associated with a greater awareness of HPV and the HPV vaccine, such as younger age, higher income, and higher educational levels, are consistent with findings from previous research on disparities in HPV vaccination intention, awareness, or vaccine uptake [29, 36, 37]. Despite the lack of interaction between race/ethnicity and political ideology, non‐White populations exhibited lower awareness compared to Whites across the political spectrum. This suggests that HCPs and policymakers should continue to focus on vulnerable populations, including race/ethnicity minorities, and those with lower income and education levels.

### 5.1. Implications for Nursing

The findings of this study highlight the need for nurses to provide targeted education and strong HPV vaccine recommendations, particularly for males, especially those with conservative political ideology, who exhibit lower levels of HPV and the HPV vaccine awareness. The persistent racial and ethnic disparities, with especially low awareness among Asian American populations, indicate the importance of delivering culturally and linguistically appropriate communication. Nurses should assess clients’ health literacy and utilize clear, accessible educational resources to address gaps among individuals with lower socioeconomic or educational backgrounds. For example, when discussing the HPV vaccine with conservative male parents, nurses may emphasize the vaccine’s role in preventing multiple types of cancer beyond cervical cancer and frame vaccination as a proactive and responsible decision that protects their child’s long‐term health.

### 5.2. Study Limitations and Strengths

While our study examined the important relationship between political ideology and HPV awareness, results should be interpreted in the context of several limitations. This study utilized the public data available, which results in lacking certain questions necessary to fully answer specific contexts. For example, this study primarily focused on HPV awareness and did not investigate the actual vaccine uptake. Although research has shown a significant relationship between HPV vaccination uptake and awareness [8, 14], there can be a gap between awareness and actual vaccination behaviors. Furthermore, our study examined adults over the age of 18, but we did not have data on whether they had a child who was vaccine‐eligible. Future studies should specifically examine the beliefs and behaviors of parents of children and adolescents or young adults. Next, the data for this study are self‐reported, subjecting respondents to social desirability biases. Respondents could answer questions in a manner that they believe is more socially acceptable or favorable, rather than providing truthful responses. Lastly, the cross‐sectional nature of our study provides a singular point‐in‐time view, limiting our ability to draw causal conclusions or track changes in HPV awareness over time. Future studies might consider a longitudinal design to better understand shifts in awareness over time across different political and race/ethnicity groups.

## 6. Conclusion

This study found that awareness of HPV and the HPV vaccine differs by political ideology, but this variation is primarily among males, with females generally being more aware of HPV and the HPV vaccine regardless of political ideology. Additionally, racial/ethnic disparities persist, with Black, Hispanic, Asian, and other minority populations showing lower awareness compared to Whites. Our findings highlight the urgent need to enhance HPV awareness in conservative males and racial/ethnic minority populations, particularly Asian Americans, by tailoring approaches to increase awareness of the dangers of HPV and the benefits of vaccination. Nursing professionals should play an essential role in delivering targeted education to improve HPV and the HPV vaccine awareness by promoting health literacy and using accessible communication strategies.

## Funding

No funding was received for this manuscript.

## Conflicts of Interest

MLK is a consultant to Merck has received investigator‐initiated research funding from Merck, administered through Purdue University. The remaining authors declare no conflicts of interest.

## Supporting Information

Additional supporting information can be found online in the Supporting Information section.

## Supporting information


**Supporting Information** Supporting Table 1. Sample characteristics (unweighted sample *n* = 3113).

## Data Availability

The data that support the findings of this study are openly available in HINTS at https://hints.cancer.gov/.
