# Correction to “Political Ideology and HPV Vaccine Awareness: Sex Differences and Nursing Implications Using the Health Information National Trends Survey (HINTS)”

**DOI:** 10.1155/nrp/9867503

**Published:** 2026-06-18

S. Jo, D. Xu, M. L. Kasting, and N. Adams, “Political Ideology and HPV Vaccine Awareness: Sex Differences and Nursing Implications Using the Health Information National Trends Survey (HINTS),” *Nursing Research and Practice*, 2026, 4233079, https://doi.org/10.1155/nrp/4233079.

In the article, there were errors in the affiliation listed below, in which multiple schools and departments were incorrectly combined into a single affiliation:

• School of Nursing, Department of Public Health, Regenstrief Center for Healthcare Engineering, Purdue University, West Lafayette, Indiana, USA

The correct author list and affiliations are shown below and have been corrected in the article:

Soojung Jo^1^, Dongjuan Xu^1^, Monica L. Kasting^2^, Nicole Adams^3^

^1^School of Nursing, Purdue University, West Lafayette, Indiana, USA

^2^Department of Public Health, Purdue University, West Lafayette, Indiana, USA

^3^Regenstrief Center for Healthcare Engineering, Purdue University, West Lafayette, Indiana, USA

We apologize for these errors.

