# Supplementary material for: Political Ideology and HPV Vaccine Awareness: Sex Differences and Nursing Implications Using the Health Information National Trends Survey (HINTS)
Source: Nurs Res Pract. 2026 Mar 28;2026:4233079. doi: 10.1155/nrp/4233079 (PMC13279362; doi:10.1155/nrp/4233079)
Supplement: Supplementary file 1 — Supporting Information Supporting Table 1. Sample characteristics (unweighted sample n = 3113). [file NRP-2026-4233079-s001.docx]

**Supplement Table 1.** Sample **c**haracteristics (unweighted sample n = 3,113)

|  | **Frequency (%) or Mean ± SD** |  |
| --- | --- | --- |
|  |  |  |
| Age (years) | 55.67 ± 16.75 |  |
| 18 - 26 | 156 (5.01) |  |
| 27 - 35 | 331 (10.63) |  |
| 36 - 45 | 401 (12.88) |  |
| 46 - 64 | 1,145 (36.78) |  |
| ≥ 65 | 1,080 (34.69) |  |
| Sex |  |  |
| Male | 1,315 (42.24) |  |
| Female | 1,798 (57.76) |  |
| Race/ethnicity |  |  |
| White | 1,934 (62.13) |  |
| Black | 402 (12.91) |  |
| Hispanic | 523 (16.80) |  |
| Asian | 152 (4.88) |  |
| Other | 102 (3.28) |  |
| Marital Status |  |  |
| Married/partnered | 1,710 (54.93) |  |
| Divorced/widowed/separated | 855 (27.47) |  |
| Single, never been married | 548 (17.60) |  |
| Education level |  |  |
| Less than high school | 188 (6.04) |  |
| High school graduate | 532 (17.09) |  |
| Some college | 913 (29.33) |  |
| College graduate or more | 1,480 (47.54) |  |
| Income |  |  |
| < $20,000 | 473 (15.19) |  |
| $20,000 to $49,999 | 814 (26.15) |  |
| $50,000 to $74,999 | 549 (17.64) |  |
| $75,000 to $99,999 | 384 (12.34) |  |
| ≥ $100,000 | 893 (28.69) |  |
| Employment status |  |  |
| Yes | 1,653 (53.10) |  |
| No | 1,460 (46.90) |  |
| Location |  |  |
| Metropolitan | 2,778 (89.24) |  |
| Non-metropolitan | 335 (10.76) |  |
| Insurance |  |  |
| Yes | 2,952 (94.83) |  |
| No | 161 (5.17) |  |
| Political ideology |  |  |
| Liberal | 959 (30.81) |  |
| Moderate | 1,063 (34.15) |  |
| Conservative | 1,091 (35.05) |  |
| HPV awareness |  |  |
| Yes | 2,012 (64.63) |  |
| No | 1,101 (35.37) |  |
| HPV vaccine awareness |  |  |
| Yes | 1,945 (62.48) |  |
| No | 1,168 (37.52) |  |

*Note:* SD: standard deviation.
